# Gastric schwannoma with atypical manifestations in an adult patient: case report and literature review

**DOI:** 10.3332/ecancer.2025.1910

**Published:** 2025-05-27

**Authors:** Yanet Isabel Carrasco Rojas, Albert Gabriel Turpo Peqqueña, Natalia Camila Zenteno Tejada, Victoria Elena Quispe Pastor, Juan Augusto Martínez San Martín, Evelyn Maria Chambilla Huellcacure

**Affiliations:** 1 Surgical Oncology Service, Carlos Alberto Seguín Escobedo National Hospital (HNCASE), EsSalud, Peral - El Filtro, S/N, Arequipa 04001, Peru; 2 Faculty of Human Medicine, Center for Research and Medical Studies (CIEM), Catholic University of Santa María, Urb San José s/n, Umacollo, Arequipa 04013, Peru; 3 Faculty of Biology, National University of San Agustin, Av Alcides Carrión s/n, Arequipa 04001, Peru; 4 Molecular Engineering Research Center (CIIM), Catholic University of Santa María, Urb San José s/n, Umacollo, Arequipa 04013, Peru; 5 Pathological Anatomy Service, Carlos Alberto Seguín Escobedo National Hospital (HNCASE), EsSalud, Peral - El Filtro, s/n, Arequipa 04001, Peru; a https://orcid.org/0009-0003-1975-5796; b https://orcid.org/0000-0002-9020-7659; c https://orcid.org/0009-0008-9331-7408; d https://orcid.org/0000-0002-0061-127X; e https://orcid.org/0009-0003-5246-8342; f https://orcid.org/0009-0001-3717-8457

**Keywords:** neurilemoma, gastric neoplasms, tumours of the gastrointestinal wall, surgical resection

## Abstract

Gastric schwannomas (GS) are rare tumours originating from Schwann cells that affect the gastrointestinal tract, posing a diagnostic challenge due to their nonspecific symptoms. We report the case of a 61-year-old female presented with gastric fullness and occasional episodes of abdominal pain. Computed tomography revealed a solid mass in the lesser curvature of the stomach, initially suspected to be a gastrointestinal stromal tumours. During surgery, an exophytic lesion was identified and confirmed histopathologically and immunohistochemically as a gastric schwannoma. GS should be considered in the differential diagnosis of gastric subepithelial tumours. Its diagnosis relies on immunohistochemistry, and surgical resection ensures effective treatment.

## Introduction

Gastric schwannomas (GS) are rare tumours originating from Schwann cells and represent about 2.0%–7.0% of benign neoplasms of the gastrointestinal tract [1, 2]. Histologically, GS are spindle cell tumours expressing the S-100 protein, characterised by a microtrabecular pattern, a peripheral lymphoid rim and occasionally germinal centers [3]. Furthermore, GS usually affects women between the fourth and sixth decades of life [1, 4]. Most cases of GS are asymptomatic because they are diagnosed incidentally on imaging studies performed for other indications [5]. However, in rare cases, they may manifest with nonspecific symptoms such as abdominal pain, gastrointestinal bleeding or palpable mass, which poses a diagnostic challenge [6]. Clinically, it is important to differentiate GS from other subepithelial neoplasms, especially gastrointestinal stromal tumours (GISTs), leiomyomas (LM) and leiomyosarcomas [7], since they can be easily confused when visualised in an upper gastrointestinal endoscopy [5]. Diagnostic confirmation is based on postoperative pathology and immunohistochemistry [8], with positive staining for S-100 protein and negative staining for c-kit, CD34, desmin or smooth muscle actin, which are distinctive features of schwannomas [9]. This report describes the case of an adult patient with an atypical gastric schwannoma, which complicated his initial diagnosis. Informed consent was obtained from the patient for the publication of this report for academic purposes and the confidentiality of the medical information used was maintained.

## Case report

**Figure 1.**
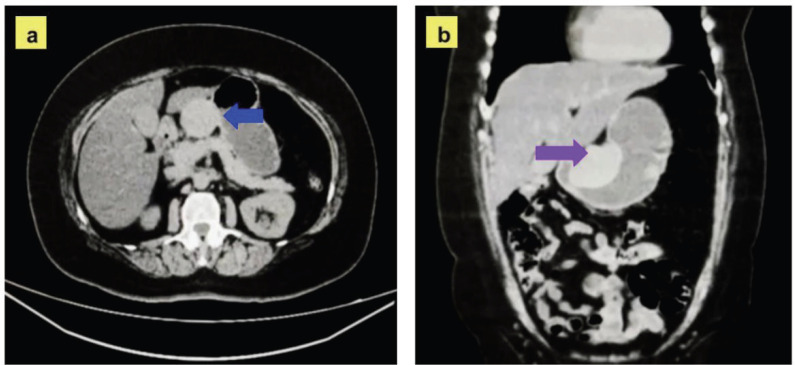
Contrast-enhanced CT with axial and coronal sections. (a): Axial section showing a solid lesion with well-defined borders, located in the lesser curvature of the stomach (blue arrow). The lesion measures approximately 64 × 49 mm, has homogeneous density and no invasion of adjacent structures. (b): Coronal section confirming the location of the lesion in the lesser curvature of the stomach (purple arrow).

A 61-year-old female patient with no history of hypertension, diabetes mellitus, smoking or allergies except to sulfonamides. Since 2019, the patient has reported a persistent feeling of gastric fullness and occasional episodes of abdominal pain. During follow-up, in January 2024, upper gastrointestinal endoscopy showed an elevated, lobulated lesion with preserved mucosa of approximately 50 mm. Subsequently, endoscopic ultrasound in February 2024 confirmed a hypoechoic image of 56 × 51 mm dependent on the muscularis propria, without internal calcifications or ductal dilatations, compatible with a subepithelial tumour. Initially, these findings suggested a GIST. Routine laboratory tests were within normal ranges, and tumour markers (Table 1) were negative. In addition, a computed tomography (CT) scan was performed, which identified a solid lesion measuring 64 × 49 mm in the lesser curvature of the stomach (Figure 1).

**Figure 2.**
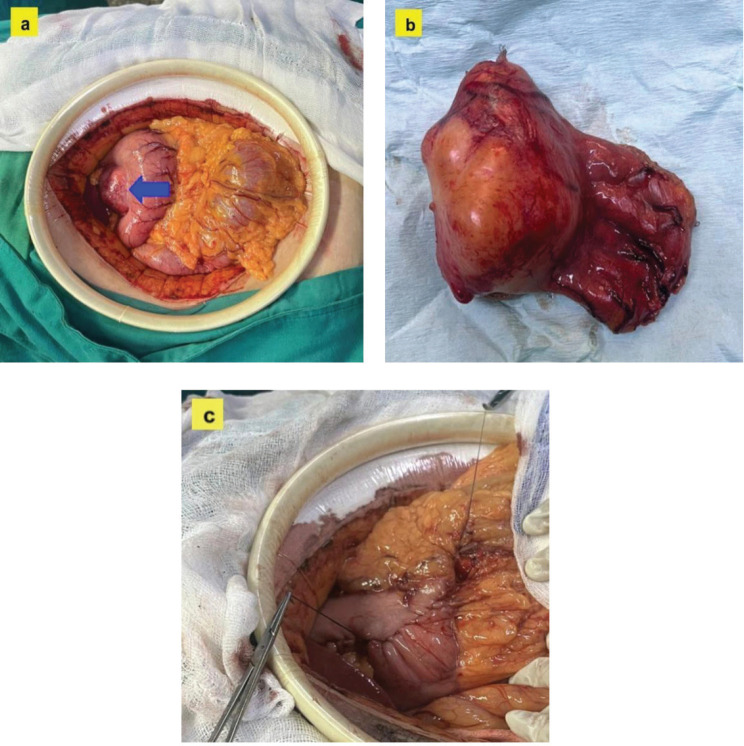
Images of the surgical procedure. (a): Intraoperative view showing the lesion in situ, displaying an exophytic tumour located in the lesser curvature of the stomach (blue arrow). (b): Image of the surgical specimen after surgical resection, showing a smooth, whitish surface with typical characteristics of a gastric schwannoma. (c): Intraoperative image illustrating the reconstruction performed by gastro-gastric anastomosis.

**Figure 3.**
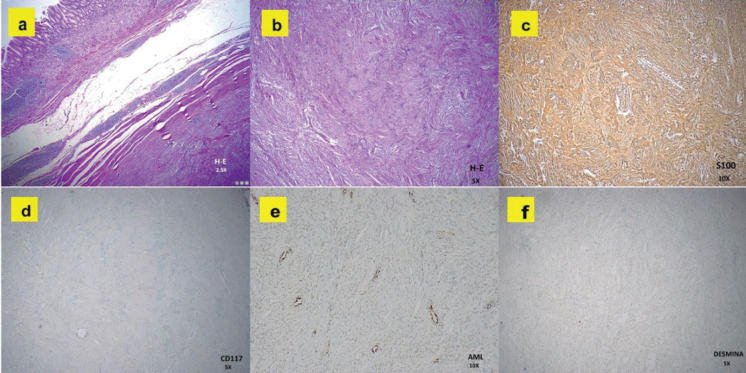
Histopathological images of the gastric tumour. (a): H&E at 2.5×: Subepithelial lesion with spindle cells in a fascicular pattern, without nuclear atypia. (b): H&E at 5×: Compact spindle cells with elongated nuclei, consistent with mesenchymal tumour. (c): S-100 at 10×: Diffuse and strong positivity, confirming neural differentiation. (d): CD117 (c-KIT) at 5×: Negative, ruling out GIST. (e): AML at 10×: Negative, excluding a lesion of smooth muscle origin. (f): Desmin at 5×: Negative, indicating the absence of myogenic differentiation.

Based on the clinical data and imaging evidence, it was decided to proceed with surgery. During the intervention, an 8 × 4 × 3 cm exophytic tumour was observed in the lesser curvature of the stomach, located 4 cm from the pylorus, with a hard consistency and a whitish surface. With these findings and a high suspicion of benign pathology, it was decided to perform a function-sparing gastrectomy: pylorus-preserving gastrectomy, preserving neurovascular structures and having a gastro-gastric anastomosis (Figure 2). The definitive diagnosis was confirmed by a histopathological and immunohistochemical study (Figure 3), reporting a gastric schwannoma (Table 2). Postoperative recovery was satisfactory, with stable vital signs and no immediate complications.

## Discussion

GS are rare submucosal mesenchymal tumours, accounting for 0.1%–3% of all gastrointestinal tract neoplasias and 1%–2% of alimentary tract mesenchymal tumours. GS arise from Schwann cells of the gastric plexus and are usually benign, unlike GISTs or LM [4, 10], which have malignant potential. Furthermore, a notable female predominance has been reported, with a female:male ratio of 4:1, in ages ranging from 40 to 60 years [1, 4, 11], and although our patient was 61 years old, her epidemiological profile coincides with the typical features of GS.

Although most of the GS are asymptomatic and are discovered incidentally during diagnostic procedures for other pathologies, with a rate ranging between 30% and 33% [4]. Pu *et al* [12], Lamb *et al* [13] and Cordera *et al* [14] reported diagnoses of GS incidentally during imaging studies for other reasons. The most common symptoms of GS include upper gastrointestinal bleeding, abdominal pain, diffuse discomfort, nausea and gastric outlet obstruction [15]. Li and Liu [16], Khan *et al* [17] and Oudad *et al* [18] reported clinical presentations with marked symptoms, such as severe abdominal pain and gastrointestinal bleeding. In our case, the patient presented nonspecific symptoms of gastric fullness and abdominal contractures, which complicated the initial diagnosis. In Zheng’s study, out of 29 cases, 5% (1 patient) reported gastric fullness as a symptom [19]. Also, in the Miettinen report, which included 1,241 patients, 1.45% (18 patients) presented gastric fullness, while 0.24% (3 patients) presented pulsatile gastric contractions [20]. Although pulsatile gastric contractions are not the same as abdominal contractures, it is relevant to note that they are present in GS.

The stomach is the most common site for GS, accounting for 60% to 70% of cases, and they usually originate mainly in the gastric body (50%), followed by the antrum (32%) and fundus (18%) [9]. GS typically presents as solitary lesions less than 5 cm in size. In Peng’s study, of 78 reported cases, 72.2% (56 patients) were located in the gastric body, specifically in the lesser curvature and middle third of the stomach [4, 21]. In this report, we found that the tumour mass was also located in the lesser curvature, coinciding with several authors (Table 3).

Endoscopy with ultrasound, as highlighted by Sanei *et al* [22], He *et al* [23] and Tang *et al* [24], is a key tool for the characterisation of subepithelial lesions. However, the diagnosis of GS is based on pathological and immunohistochemical findings, and several authors highlight S100 as a specific marker with 97.6%100% positivity (Table 3) [4]. GS is also positive for vimentin, but negative for c-kit, CD34 and CD117, which contrasts with GIST, and are also negative for desmin and smooth muscle actin, unlike LM [15, 25, 26]. In our case, the diagnosis was confirmed by immunohistochemistry, with positivity for S-100 and negativity for c-kit, CD34, desmin and smooth muscle actin (Table 2).

The main treatment of GS is surgical resection, and it is important to ensure the complete removal of the tumour [27]. Function-sparing gastrectomies, such as segmental or subtotal, are the most commonly used approach for large tumours or those located in complex areas, as reported by Pu *et al* [12], Kostovski *et al* [28], He *et al* [23] and Lomdo *et al* [29]. This approach ensures negative margins and minimises recurrence. On the other hand, endoscopic resection is used for smaller tumours or those without suspicion of deep invasion, as reported by Cruz Centeno *et al* [30], Tang *et al* [24] and Sorial *et al* [31], due to its lower morbidity and rapid recovery.

## Conclusion

Our case report highlights the importance of including GS in the differential diagnosis of gastric subepithelial tumours, particularly in patients with nonspecific symptoms. Definitive diagnosis depends on histopathological and immunohistochemical studies, with positivity for S-100 and negativity for c kit and CD34. Complete surgical removal, with a function-sparing approach, ensures effective treatment and favourable outcomes.

## Conflicts of interest

Our authors have no competing interests, and the contents of this manuscript have not been published elsewhere. There are no conflicts of interest to disclose.

## Funding

No external funding or funding by any author has been received for this study.

## Informed consent

Informed consent was obtained from the patient for the publication of this report for academic purposes and the confidentiality of the medical information used was maintained. A copy of the written consent is available for review by the Editor-in-Chief of this journal.

## Author contributions

The original manuscript was authored and approved by all contributing authors.

**Yanet Isabel Carrasco Rojas:** Review of intellectual content, data collection, preparation of photographs and approval of the version sent to the editorial process.

**Albert Gabriel Turpo Peqqueña:** Preparation of the document from its conception, design, complete writing and final modifications, acquisition of information, critical review of intellectual content and approval of the version sent to the editorial process**.**

**Natalia Camila Zenteno Tejada:** Bibliographic review, contributions to the intellectual content and approval of the version sent to the editorial process.

**Victoria Elena Quispe Pastor:** Bibliographic review, contributions to the intellectual content and approval of the version sent to the editorial process.

**Augusto Martínez San Martín:** Preparation of photographs, bibliographic review, contributions to the intellectual content and approval of the version sent to the editorial process.

**Evelyn Chambilla Huellcacure:** Preparation of photographs, bibliographic review, contributions to the intellectual content and approval of the version sent to the editorial process.

## Figures and Tables

**Table 1.** Auxiliary examinations of the patient during hospitalization.

Date	07/02/2024
CA 19-9	0.6 U/mL
AFP	1.86 ng/mL
CEA	2.6 ng/mL

**Table 2.** Histopathological and immunohistochemical examination.

Staining	06/06/24
S-100 protein	Positive
c-kit (CD117)	Negative
CD34	Negative
Smooth muscle actin (SMA)	Negative
Desmin	Negative

**Table 3.** Cases reported in the literature of gastric schwannoma, 2018–2024.

Author, country and year	Sex and age	Clinic	Diagnosis	Site and size of the GS	Treatment
Pu and Zhang, China, 2020 [12]	Male 30	Right upper quadrant abdominal discomfort	Positive for S100 and GFAP, negative for CD34 and CD117	Gastric body 5.1 × 4.4	Laparoscopic wedge resection of the stomach
Lamb *et al*, EEUU, 2024 [13]	Female 67	Incidental finding of mass during regular medical checkup	Positive for S100	Gastric body 6.9 × 6.0	Surgical technique not mentioned
Cordera *et al*, México, 2019 [14]	Male 68	No weight loss, changes in diet or appetite; normal gastrointestinal function	Positive for S- 100 and SOX10, and negative for CD117 and DOG-1	Lesser curvature of the stomach 5.1 × 2.1	Laparoscopic resection
Li and Liu, China, 2022 [16]	Female 64	Dizziness and headache for 3 day	Gastric schwannoma with nuclear division of < 5/50 by highpower field. Positive for S- 100, and negative for CD117, CD34, DOG- 1, actin, and desmin	Fundus of stomach 4.7 × 4.4	Proximal subtotal gastrectomy, radical lymphadenectom y, esophagogastric anastomosis.
Khang *et al*, EEUU, 2022 [17]	Female 68	Abdominal pain, nausea, vomiting, belching	Positive for S-100, negative for CD117, smooth muscle actin, CD34	Gastric body 4.2	Gastrectomy with Roux-en-Y reconstruction
Oudad *et al*, Marruecos, 2022 [18]	Female 67	Abdominal discomfort for 6 months	Positive for S100	Cardia of the stomach 9	Laparotomy with atypical midline gastric resection with gastrogastric anastomosis
Al-Zawi *et al*, Reino Unido, 2022 [21]	Female 79	Abdominal pain with history of hypercholesterol emia and hypertension; previous angioplasty	Positive for Vimentin, S100 and glial fibrillary acidic protein (GFAP). Negative for c-KIT, DOG1, Desmin, H- caldesmon and CD34	Greater curvature of the stomach. 3 × 2.8 × 2	Laparoscopic sleeve gastrectomy and resection of the submucosal mass at the greater curvature.
Sanei *et al*, Irán, 2018 [22]	Female 28	Abdominal pain for several weeks, recurrent vomiting, hematemesis, and severe anorexia	Positive for S-100 diffusely in the nucleus and cytoplasm. Negative for CD117, CD34, KI67, actin, chromogranin, and desmin	Antrum of stomach 5 × 6	￼Open surgery, subtotal gastrectomy with Roux-en-Y gastrojejunostom y
He *et al*, China, 2022 [23]	Female 28	Two months previously, epigastric discomfort and abdominal fullness	Gastric schwannoma with Ki-67 protein <3%, positive for S-100 and SOX10, negative for desmin, DOG-1 (-), smooth muscle actin, CD34, CD117 and P53	Stomach Antrum 4.5 × 4	Full-thickness endoscopic resection with endotracheal intubation, additional laparoscopic surgery for large defect, difficult endoscopic closure and tumor removal via esophagus.
Teng *et al*, China, 2020 [24]	Female 79	Loss of appetite, intermittent vomiting, gastric contents, one month	Gastric schwannoma with Ki-67 protein <5%, positive for S-100 and SOX10, negative for CK7, CK20, CK, villin, CDX-2, CD117, CD34, SMA, desmin and cerbb-2	Between the body of the stomach and the angular notch 4.5 × 6	Subtotal laparoscopic gastrectomy
Kostovski *et al*, Macedoni a, 2024 [28]	Male 68	Lower abdominal discomfort for 6 weeks, no gastric symptoms, abdomen without tenderness or palpable masses	Gastric schwannoma with Ki-67 protein <1% and positive for S-100	Greater curvature towards the antral region. 5.7	Supraumbilical laparotomy, with tangential excision of the gastric wall and total removal of the tumor.
Lomdo *et al*, Marrueco s, 2020 [29]	Female 73	Intermittent abdominal discomfort	Positive for S100	Greater curvature of the gastric body 8 × 8 × 6	Laparoscopic wedge resection of the stomach
Cruz Centeno *et al*, Puerto Rico, 2021 [30]	Female 68	Incidental finding during hernia evaluation; asymptomatic	Positive for S-100, negative for CD117 and DOG1	Gastric fundus 4.4 × 4.3 × 3.2	Laparoscopic wedge resection of the stomach
Sorial *et al*, EEUU, 2024 [31]	Female 84	Abdominal pain, clinically stable	Gastric schwannoma with Ki-67 protein < 2- 3%, positive for S-100 and SOX10, negative for CD34, CD117 DOG1, desmin and smooth muscle actin	Greater curvature of the gastric body 4	Diagnostic laparoscopy with stapled gastric wedge resection
Yanagawa *et al*, Japón, 2020 [9]	Female 66	Incidental finding on X-ray after laparoscopic cholecystectom y	Positive for S-100 and negative for ckit, CD34, desmin or smooth muscle actin	Greater curvature of the stomach 5.2	Laparoscopic wedge gastrectomy, complete resection, negative surgical margin
Lu and Zhao, China, 2021 [32]	Female 45	Incidental finding of mass during regular medical checkup	Positive for S100	Greater curvature of the gastric body 2.0 × 1.8	￼Total endoscopic resection, pursestring suture
Mohamad *et al*, Malasia, 2020 [33]	Male 77	Patient with Parkinson's disease, hypertension, diabetes mellitus, on medical treatment, worsening abdominal discomfort, early satiety	Positive for S100 and negative for CD117, DOG1, SMA, EMA, CD34, BCL2, desmin and CK AE1/AE3	Posterior surface of stomach 11 × 10 × 9.9	Partial gastrectomy with roux-en-y bypass procedure
Majdoubi *et al*, Marruecos, 2024 [34]	Male 50	Persistent postprandial gastric pain for 1 month, melena, anorexia and asthenia	Positive for S100	Greater curvature of the gastric body 3.5 × 3.2 × 3.1	Atypical gastric resection by laparoscopic approach
Pais *et al*, Portugal, 2024 [35]	Male 63	Epigastric pain. History of hypertension and dyslipidemia.	Gastric schwannoma expression of CD34 and SOX10, without immunostaini ng for S-100 protein, Ml actin, desmin, STAT6, DOG1 or c-kit	Greater curvature, at the transition between the body and the gastric antrum 2.2 × 1.8	Atypical laparoscopic gastrectomy
Fugărețu *et al*, Rumania, 2023 [36]	Male 56	Incidental finding of mass during ultrasound.	Positive for S100	Lesser curvature of the stomach. 5 × 4.2 × 3.1	Subtotal laparoscopic gastrectomy
Albshesh *et al*, Israel, 2020 [37]	Female 40	Incidental finding of mass during regular medical checkup	Positive for S100	Greater curvature of the stomach 3.7 × 4.8	Complete gastric resection
